# Consumption-Evoked Emotions from Meat and Plant-Based Meat Alternatives and Their Influence on Meat Reduction

**DOI:** 10.3390/foods15122179

**Published:** 2026-06-17

**Authors:** Stergios Melios, Niamh Harbourne, Declan Bolton, Emily Crofton

**Affiliations:** 1 Teagasc Food Research Centre, Ashtown, D15 DY05 Dublin, Ireland; declan.bolton@teagasc.ie (D.B.); emily.crofton@teagasc.ie (E.C.); 2 Institute of Food and Health, School of Agriculture and Food Science, University College Dublin, D04 V1W8 Dublin, Ireland; niamh.harbourne@ucd.ie

**Keywords:** Consumption-Emotion-Update framework, theory of constructed emotion, practice theory, sustainability, meat alternatives, meat reduction, sensory

## Abstract

Growing concerns about health and sustainability have increased interest in meat consumption and reduction. Emotions evoked during the consumption of meat and plant-based alternatives can significantly shape choices. This review examines theoretical and empirical evidence on emotions experienced during the consumption of meat and plant-based alternatives. Drawing on practice theory and the theory of constructed emotion, it proposes a mechanism through which emotions evoked during consumption influence subsequent decisions to consume or reduce meat. This narrative review first defines emotions and examines their role in meat consumption and reduction and then evaluates studies on emotions elicited by tasting meat and plant-based alternatives. The findings suggest that discussions around meat consumption evoke emotions of both positive and negative valence, which may create cognitive dissonance. However, during actual meat consumption, emotions of positive valence are most commonly reported. In contrast, plant-based alternatives tend to elicit emotions of negative valence, particularly when their sensory characteristics differ substantially from those of meat. This review hypothesises that emotions experienced during meat consumption generate prediction errors that update the brain’s internal model, thereby reinforcing or adjusting subsequent attitudes and choices. This mechanism is conceptualised as the Consumption-Emotion-Update (CEU) framework. Understanding how consumption-evoked emotions shape food choice behaviour may inform strategies aimed at promoting healthier and more sustainable diets.

## 1. Introduction

In the context of growing concerns about sustainability and health, meat consumption has become an important topic, with future policies likely to have significant implications for meat production and trade [1,2]. This is particularly evident in the ongoing debate regarding the dietary greenhouse gas emissions associated with meat-centred diets and how they compare to vegan or lacto-ovo-vegetarian diets [3,4,5].

Consequently, several alternatives have been proposed to partially replace meat in human diets, and their acceptance by consumers is currently being investigated [6,7,8]. The degree to which these alternatives resemble conventional meat has been identified as a key factor influencing consumer acceptance [9,10]. While several meatless products have already achieved a high level of sensory and experiential similarity to meat [11], others still fall far behind [6,12]. Among meat alternatives, those derived from plant sources have gained the greatest consumer acceptance [13] and have also been widely commercialised [14]. However, despite their initial rapid growth, the plant-based meat alternative (PBMA) market has slowed significantly [15].

Meat consumption is a topic of broad interest, encompassing psychological, anthropological, environmental, and moral dimensions, and it is deeply embedded in society [16,17,18,19,20]. To predict the acceptability and eventual choice of PBMAs, measuring preferences alone may not be sufficient. Emotions provide additional and valuable insights in this regard [21,22]. The emotional profile of a food has been shown to distinguish between similar products, even when liking scores are comparable [23]. Thus, examining both a product’s sensory characteristics and the emotions evoked during its consumption can provide a deeper understanding of the overall consumption experience and its influence on consumer choice [24]. It is important to note that in the context of food consumption, emotional responses are primarily shaped by the specific sensory attributes of an individual product rather than by the broader product category to which it belongs [25].

The emotions elicited during food consumption are influenced by a combination of factors, including the sensory characteristics of the product, the broader food category to which it belongs, and individual consumer traits such as age, gender, behavioural tendencies, physiological state, and pre-existing mood or feelings. In addition, the specific moment and circumstances of consumption can further shape emotional responses [21,23,26,27]. As most studies that assess emotions elicited by food have been conducted under controlled conditions [28], it is important to note that the context in which food is consumed can also have a significant influence on emotional experiences. This context may encompass factors such as dining location, packaging, product name, and information about a food’s health benefits or production methods [21,23]. It is interesting that although emotions linked with the eating context are often based on remembered experiences as opposed to those occurring during consumption itself, they could be particularly influential as they encompass elements of the entire eating experience, including post-eating impressions and evaluations [21,28].

As mentioned above, the literature on meat consumption addresses multiple dimensions, while research on the environmental impacts of meat production continues to expand. At the same time, considerable attention has been devoted to the development of PBMAs as a potential strategy for reducing meat consumption and production, as well as to understanding the factors behind the recent slowdown in the growth of the PBMA market. Although emotions experienced during the consumption of meat and its alternatives have been shown to play a key role in product acceptance, research combining existing knowledge on consumption-evoked emotions and their influence on food choice behaviour remains limited. While several review articles sit at the intersection of food and emotions, they mainly focus on methodological aspects or on linking emotions to choice, without addressing the behavioural mechanisms involved. They also rarely consider how emotions are formed and change over time through experience and socio-cultural context. As a result, emotions are often treated as correlates of choice rather than dynamic processes embedded in broader psychological and social mechanisms shaping consumption behaviour.

Therefore, this narrative review aims to provide insight into the relationships between emotions, the consumption of meat and PBMAs, and meat reduction, based on practice theory and the theory of constructed emotion. Rather than providing a systematic or exhaustive assessment of the literature, it presents an integrative perspective on the available evidence and should be interpreted accordingly. The review begins by examining what an emotion is, and then explores the role of emotions in meat consumption and reduction. It subsequently reviews studies examining the emotional response of consumers during the actual tasting of meat and PBMAs. While many other psychological factors influence meat consumption and reduction, this narrative review focuses specifically on emotions and considers other mechanisms only insofar as they relate to emotional responses. Therefore, the primary aim of this review is to explore how emotions elicited during the actual consumption of meat and PBMAs influence intentions and decisions related to meat consumption or reduction. In addition, this review examines how these emotions interact with other aspects such as consumption context, liking and cognitive dissonance. Based on the available evidence, rather than drawing definitive conclusions, this review proposes a conceptual model that could guide future research and be further examined and empirically validated. By advancing our understanding of the role of consumption-evoked emotions, this review contributes to ongoing discussions on meat reduction and the potential of plant-based alternatives in replacing a portion of meat in human diets, thereby supporting the transition toward healthier and more sustainable food systems.

## 2. What Is an Emotion?

It has been suggested that emotions can be viewed as forms of practical engagement with the world [29,30]. From this perspective, emotions are understood as emerging from embodied dispositions that are shaped by social contexts and, therefore, are inherently influenced by cultural and historical circumstances. Viewing emotions as practices highlights their dynamic and situated nature, emphasizing that they arise through interactions between individuals and their environments [29].

There is often confusion between the terms mood and emotion [28]. Mood tends to have a longer duration and lower intensity, whereas emotions are typically short-lived and associated with specific stimuli. Emotions can occur consciously or unconsciously, and the identification of these stimuli or emotions themselves can sometimes be challenging [21]. Barrett, drawing on neuroscience, shows that an emotion is the brain’s interpretation of bodily sensations in relation to the surrounding environment [31]. According to this perspective, emotions are not fixed entities but dynamic constructions. Considerable variation exists within each emotion category, while instances belonging to the different categories can share similarities. Moreover, the workings of each system cannot be studied in isolation but must be understood holistically in the momentary state of the brain, body, and environment, and emotional episodes have functional features beyond those of physical states alone [32]. Barrett further explains that through past experiences and collections of embodied, whole-brain representations, the brain continuously generates predictions about incoming sensory information, and the best actions to address impending events, and their consequences for allostasis. These predictions, together with prediction errors, shape perceptions and experiences [33]. Additionally, it should be noted that theories of basic emotions, which propose that each emotion is supported by a discrete and independent neural system, do not fully account for empirical findings from affective neuroscience. In contrast, valence and arousal provide a fundamental substrate for all affective states, upon which cognitive processes are layered to interpret and refine emotional experience [34].

Therefore, emotions cannot be understood as hardwired biological givens supporting an essentialist view. Instead, they are the result of cultural processes and learned experiences, cognitively and socially constructed over time, and can only be fully understood in their social and cultural context. Additionally, the brain predicts both sensory events and the body’s relative needs through allostasis, preparing the body accordingly. When prediction errors occur, the brain adjusts its model to better align with experience.

## 3. Review Methodology

Section 4 and Section 5 present a narrative review of the literature identified using search terms such as “meat”, “pork”, “beef”, “chicken”, “lamb”, “cured”, “consumption”, “reduction”, “abstinence”, “vegetarian*”, “flexitarian*”, “vegan*”, “emotion*”, “affect*”, “valence”, “arousal”, “guilt”, “disgust”, “pride”, “feeling*”, “identity”, “tasting”, “liking”, “pleasure”, “food choice*”, “acceptance”, “plant-based”, “meatless”, “substitute*”, “alternative*”, “PBMA”, “cognitive dissonance”, and “ambivalence”, as well as their combinations. Searches were conducted across the Scopus, PubMed, Google Scholar, and Web of Science databases. There was no publication date restriction applied to the studies. While similar search strategies were applied for both sections, the inclusion and exclusion criteria differed between them. Articles were searched in September 2023, and additional searches were conducted in November 2025. All records identified through the searches were screened for relevance based on title and abstract. Potentially relevant articles were then assessed in full text and selected according to the inclusion and exclusion criteria. As this was not a systematic review, no formal quality assessment or risk-of-bias evaluation was conducted.

The studies reviewed in Section 4 explicitly addressed emotions related to meat consumption or reduction, thereby providing a conceptual background for Section 5. Consequently, Section 4 excluded studies that examined general opinions, attitudes, or psychological constructs not directly related to emotions (e.g., food neophobia or meat attachment), studies that investigated emotions elicited by live animals or images of animals rather than meat products, and studies involving animal testing or behavioural manipulation techniques. Studies involving actual product tasting were also excluded.

Section 5 focussed on studies examining the consumption of meat and PBMAs assessed exclusively through direct tasting experiences. Studies examining the influence of product presentation, sensory characteristics, or information cues on emotional responses were included. Studies investigating strategies to reduce meat consumption (e.g., warning labels) or promote PBMA intake (e.g., nudging, cognitive reappraisal) using only packaging or images, without an actual tasting component, were excluded as they were considered outside the scope of this review. Additionally, studies focusing on general opinions, attitudes, or psychological constructs not linked to a specific consumption experience were likewise excluded.

However, it should be noted that this narrative review was not intended to provide an exhaustive account of the literature and may not capture all relevant studies. Rather, it aimed to provide an exploratory synthesis of the evidence concerning how emotions elicited during the actual consumption of meat and PBMAs influence intentions related to meat consumption or reduction. Moreover, the currently available evidence remains limited and does not support robust generalizations about the emotions evoked by meat. Instead, the findings are used to inform a proposed conceptual mechanism, which may be incorporated into future research and intervention design.

## 4. Emotions, Meat Consumption, and Reduction

As much of the current discourse centres on meat reduction and the potential of PBMAs as suitable replacements, this section examines how emotions are discussed in the literature in relation to meat consumption and reduction. As noted, this section does not aim to provide a systematic or exhaustive review but rather an integrative perspective on the available evidence, intended to frame the topic and support the hypothesis presented in Section 5.

Meat consumption is associated with emotions of both positive and negative valence. While a shift towards lower meat consumption is often associated with more conscious eating habits and greater enjoyment, pleasure remains a common emotion linked to meat consumption [35]. The maintenance of those emotions, of positive valence, serves as a negative predictor of reduced meat intake, whereas inducing emotions of negative valence does not necessarily predict reduction [36]. People eat animals while also loving them, a phenomenon commonly referred to as the “meat paradox” [37,38]. When individuals become aware of this paradox, they experience an aversive cognitive conflict. To resolve it permanently, they must refrain from eating meat [39]. Additionally, according to the theory of cognitive dissonance, individuals are motivated to maintain internal consistency among their beliefs, attitudes, knowledge, and values. Therefore, they experience psychological discomfort, when inconsistencies arise. This creates a drive to reduce the dissonance. The degree of inconsistency shapes the intensity of this discomfort; the greater the dissonance, the stronger the motivation to resolve it [40]. The act of actually eating meat, though, has proven to trigger psychological processes that help regulate the emotions of negative valence associated with consuming animals [38] and therefore reduce the dissonance. This finding is particularly important and should be kept in mind for the discussion in Section 6.

The interplay between emotions and meat consumption plays an important role in society. Meat has been described as deeply embedded in our affective economies, functioning as a symbolic food that contributes to the creation of shared culture and national identity, while also serving as a political object through which different societal values and viewpoints are expressed and defended [41]. Eating meat can foster a sense of shared identity by linking people emotionally to an imagined national value or relevant characteristics, often in a sentimental way [42]. Other personal and social identities are also associated with meat. For instance, are more likely to consume animals, individuals who place a high value on masculinity, enjoy eating meat, do not perceive meat consumption as a moral issue, and find dominance and inequality acceptable [38]. From this perspective, meat consumption represents more than a dietary behaviour. It reflects aspects of personal identity, social belonging, and the ways individuals relate to the social world around them, all of which are closely intertwined with affective and emotional processes.

As a consequence, pressure to change meat consumption practices may be perceived as a threat to preferred and culturally established habits [41]. A nice example is “Meat Out Day” in Colorado, an initiative encouraging residents to abstain from eating meat for one day. The campaign provoked both support and opposition, with individuals expressing their agreement or disagreement through emotions of either positive and negative valence, respectively [43]. Another example can be nudging, which has been proposed as an effective strategy for increasing the selection of plant-based options in catering settings. When viewed through the lens of emotion, consumers who typically choose meat-based dishes experience lower satisfaction and enjoyment when restricted to meat-free alternatives, leading to a decline in positive affect [44]. Moreover, climate anxiety, defined as “increased emotional, mental, or somatic distress in response to dangerous changes in the climate system”, has been identified as a positive predictor of meat reduction intentions [45,46]. Therefore, reducing meat consumption can be controversial, as some consumers perceive it as a threat to their culture and sense of belonging, while others view it as a moral issue concerning our responsibility towards animals and as an environmental concern [41]. Negative emotions, especially triggered by central policies and depending on what they are associated with, can either reinforce attachment to meat or foster its rejection.

Nevertheless, although much of the literature discusses meat reduction as a result of central policies, evidence dating back to 2000 suggests that this trend had already begun, driven by several factors. Some of the most important drivers included feelings of disgust and repulsion, or aversion to eating meat, explained by the way meat is produced and processed in modern agriculture and industry, its animal origin, the food culture associated with meat consumption, and the perception of meat as unhealthy. Consequently, consumers had already started restructuring their meals, with meat no longer occupying the central place [47]. This reduction is closely tied to emotions. Consumers tended to use emotional rather than neutral language to describe meat production and processing, often expressing disgust similar to that reported in relation to meat’s origin in living animals [47]. Another factor that has been studied in relation to the elicitation of negative emotions is anthropomorphism. While anthropomorphizing other foods can sometimes have a positive effect on purchase intention, when applied to meat it often evokes emotions of negative valence, such as guilt [48]. As such, meat reduction is not only a result of top–down initiatives but is also shaped by bottom–up emotional processes.

The main emotion of negative valence associated with meat consumption is disgust. Disgust, intertwined with visceral reactions and moral aversion, plays a central role in the discussion of meat reduction [49,50]. While disgust often involves withdrawal or avoidance, it can also coexist with curiosity or fascination, highlighting how these emotions are embedded in social and relational contexts [49]. Disgust towards meat is generally linked to lower meat-related ambivalence [39]. Although research does not fully support an emotivism perspective, according to which moral opposition to meat consumption does not cause disgust, but rather pre-existing feelings of disgust make individuals more likely to develop negative moral judgments about eating meat [51], it is possible that both processes coexist. Moral judgments may not only concern the fact that meat is derived from animals but also its production and processing methods. Considering the theory of constructed emotion, and through the lens of practice theory, disgust related to meat consumption can be shaped by social norms, stigma, and moral judgments. People may therefore react differently to the same stimulus depending on what it represents to them.

Omnivores tend to experience lower levels of disgust and guilt and hold stronger justifying beliefs about meat consumption compared with those who do not consume animal products [52]. They use meat-related dissonance reduction strategies to a greater extent than groups with other dietary profiles, such as denying animal suffering and employing dichotomization [53]. They may also resolve the conflict between enjoying meat and caring about animal welfare by perceiving animals as unworthy or unfeeling [37]. Cognitive dissonance is also found among meat reducers. It has been reported that for this group, the conflict between doing the “right thing” and seeking pleasure leads to emotions of negative valence, such as guilt, disappointment, and regret, and prompts justifications aimed at maintaining balance or accommodating others’ preferences [54]. For example, reducers may experience feelings of guilt during consumption, which they associate with reduced enjoyment or later reflections on their choices. In this way, adhering to normative values and goals can undermine hedonic enjoyment [54]. Beyond dietary profiles, there are also distinct consumer subgroups within omnivores who handle meat-related issues differently. For example, indifferent consumers do not experience conflicting thoughts; struggling consumers experience cognitive conflict accompanied by emotions of negative valence; and coping and strategically ignorant consumers both find ways to manage these conflicts, either by changing their behaviour or by ignoring the issue altogether [55]. It seems that emotions, both across dietary groups and within them, shape how decisions to consume or reduce meat are made, with actual consumption subsequently influencing future decision-making processes.

Similarly, several other individual characteristics influence meat consumption and reduction, as well as their association with emotional states. An analysis based on the Big Five personality traits revealed that openness to experience had a direct and significant negative effect on meat consumption, whereas extraversion had a direct and significant positive effect. The effects of neuroticism, conscientiousness, and extraversion on eating styles were consistent with those observed for other food categories. However, only external eating was significantly and positively associated with meat consumption. In addition, extraversion had a significant positive indirect effect on meat consumption, while conscientiousness had a significant negative indirect effect [56]. In another study examining the Big Five traits, meat consumption was found to be negatively associated with openness and emotional stability, and positively associated with extraversion [57].

Emotions play a central role in shaping the effectiveness of different messaging strategies aimed at reducing meat consumption. For example, persuasive messages that evoke disgust have been shown to be more effective than health-oriented messages and are at least as effective as messages emphasizing moral concerns, such as animal welfare [58]. Another study compared a “warm-glow” message (stating that reducing meat consumption will make one feel good) with a “cold-prickle” message (stating that not reducing meat consumption will make one feel bad). “Warm-glow” messages were positively associated with consumers’ intentions to reduce meat consumption, even more strongly than perceived sustainability and health benefits messaging. However, this type of messaging was less effective in increasing intentions to consume meat substitutes [59]. In addition, meat-shaming messages tend to elicit shame and other emotions of negative valence, which in turn reduce purchase intentions [60]. Vegans and vegetarians generally report higher levels of disgust towards meat, yet both them and omnivores experience increased disgust after reading descriptions of poor hygienic conditions in meat production. Moreover, consumers who do not morally disengage from their behaviour that might be considered harmful experience heightened disgust when exposed to information about animal cruelty in meat production [39]. Finally, positive cognitive reappraisal, the reinterpretation of a stimulus to positively change its meaning, has been shown to enhance positive emotional responses toward alternative proteins, which in turn increases the desire for such products [61]. These findings provide evidence that interventions can be designed to alter emotional responses to meat consumption and thereby influence choices to consume or reduce it.

Evidence summarised in this section suggests that emotions surrounding meat consumption and reduction are not fixed reactions to meat itself. Rather, they are context-dependent constructions shaped by learned experiences and social practices. Drawing on Bourdieu’s practice theory, these emotions emerge through embodied practices involving the body and mind, material artefacts, the environment, and interactions with other people. Through repeated cultural exposure, individuals develop embodied expectations about what meat represents, how it should feel to consume it, and how it relates to identity, values, or social belonging. From the perspective of the theory of constructed emotion, the brain continuously predicts both the sensory and, consequently, emotional experiences associated with eating or avoiding meat, preparing the body accordingly on the basis of past experiences and social context. As presented above, moral concerns, disgust, climate anxiety, or social pressure may coexist and conflict with positive affective associations, such as pride, pleasure, or a sense of masculinity. These competing emotional expectations can create prediction conflicts that influence attitudes toward meat and ultimately shape consumption behaviour.

## 5. Emotional Response to the Consumption of Meat and Plant-Based Meat Alternatives

This section of the review will focus on the consumption experience of meat and PBMAs, assessed exclusively through direct tasting. Studies in which meat was a minor component within complex dishes were excluded. The primary aim of this section is to examine the emotional responses elicited by direct product consumption or evaluation without interpreting their broader meaning or implications. This descriptive synthesis provides the empirical foundation for Section 6, in which we propose a mechanism explaining how emotions elicited during actual consumption of meat and plant-based meat alternatives may influence intentions related to meat consumption or reduction.

Most of the identified studies investigating explicit emotions employed methods such as Check-all-that-apply (CATA) or Rate-all-that-apply (RATA), combined with either predefined or consumer-generated emotional lexicons, including EsSense, EsSense25, and the EmoSensory Wheel. In some studies, consumers were provided with the EsSense Profile lexicon to assist them in developing their own emotional vocabularies. Moreover, an emotional lexicon introduced by Orr et al. (2023) [62], designed specifically for meat and plant-based burger patties, was also used. Compared with the EsSense Profile, consumer-generated lexicons contained a significantly greater number of negative terms. This observation should be considered in future research, as it suggests that consumers may require a broader range of negative terms beyond those offered by predefined lexicons to accurately describe their emotions in the context of meat consumption. The decision to focus on emotions of either positive or negative valence ultimately depends on the specific objectives of the study; for instance, the examination of food product deterioration [23] might require a questionnaire with more negative emotions. Studies exploring consumers’ emotional response to meat products, either explicitly or implicitly, are summarised in Table 1 and Table 2, respectively, while studies including plant-based alternatives, either alone or in combination with meat products, are presented in Table 3.

### 5.1. Explicitly Measured Emotional Responses to Meat Products

In all the studies, the emotional response to meat consumption was predominantly positive while not many differences in terms of arousal were reported. Emotions such as interest, calmness, activeness, security, pleasure, contentment, and enthusiasm were consistently among the highest-rated emotions with respect to beef. Less frequently, emotions of negative valence were reported such as disgusted, bored, guilty, worried, aggressive, and wild [63,64,67].

#### 5.1.1. Type of Product

Different beef cuts were able to evoke varying emotions. For instance, the rump cap cut tended to elicit higher scores for emotions of positive valence, while the outside flat cut was associated with emotions of negative valence [64]. When it comes to meatballs, the most commonly reported emotions were of positive valence, such as satisfaction, enjoyment, pleasant surprise, and desire. Even when recycled water was used in the preparation of beef meatballs, the emotions of disgust and fear were experienced the least [69]. Meat burgers, on the other hand, were predominantly linked with emotions of positive valence, with contentment being the primary emotion, followed by happiness, merriment, and pleasantness [66].

Emotion profiling has also been employed to distinguish between meat products derived from various breeds or processes. Notably, Iberian dry-cured ham, renowned for its quality, even if it was served without information about its origin, primarily evoked emotions of positive valence such as intensity, pleasantness, desirability, festiveness, liveliness, tradition, satisfaction, authenticity, and naturalness. In contrast, lower reputed varieties like Curado and Serrano tended to elicit mostly emotions of negative valence, including dissatisfaction, unpalatability, thirst, indifference, disgust, and ordinariness. The dominant emotions associated with Iberian dry-cured ham were authenticity, pleasantness, and intensity, while for Serrano and Curado, the dominant emotions were ordinariness, indifference, intensity, and dissatisfaction [71]. However, for rib-eye steaks from different biological types, no significant differences were observed regarding the emotional response of consumers [67].

#### 5.1.2. Additional Ingredients

The effect of adding other ingredients, either to enhance flavour or to reduce salt content, has also been investigated. In the case of chicken, the use of spice blends or chicken breast was associated with emotions of positive valence, such as pleasantness, desirability, festiveness, satisfaction, refreshment, comfort, bliss, relaxation, and liveliness. On the other hand, plain chicken (without seasoning) or chicken obtained from other parts like the butt, skin, head, and thigh was linked to emotions of negative valence, such as annoyance and abhorrence [70,74]. A study examined salt reduction in burgers in combination with seaweed addition. Overall, consumers associated burgers with emotions such as good, pleasant, satisfied, and warm. Reduced-salt samples containing 0.5–1% seaweed were perceived more positively than plain salty samples or those containing only seaweed and no salt. Unsalted burgers, on the other hand, were associated with more emotions of negative valence than salted ones, such as bored, mild, and tame. A higher level of seaweed addition (2.5%) was linked to slightly higher citations of adventurous, but also higher citations of aggressive and wild emotions [78]. However, when roasted chicken with varying KCl/NaCl replacement levels was evaluated for different emotions (i.e., good, happy, interested, pleased, satisfied, unsafe, and worried), no significant differences were found across treatments [77].

### 5.2. Implicitly Measured Emotional Responses to Meat Products

Neutral facial expressions were the most commonly observed during the consumption of beef patties, grilled pork, and smoked ham [79,80,81].

#### 5.2.1. Consumer Characteristics

It is interesting to note that older people tended to display more neutral and disgust emotions on their faces compared to younger ones. In contrast, younger individuals tended to exhibit a broader range of emotions, including happiness, sadness, and fear. Additionally, younger people appeared to be less happy when consuming hard beef patties, while older individuals expressed more anger when consuming medium-hard and hard patties [80]. Moreover, Mena et al. (2023) [80] reported that young consumers eating soft and hard patties had a higher heart rate than their older counterparts.

**Table 2:** Studies on emotions elicited by consumption of meat, measured implicitly.

	Product	Technique/Outcome Measured	Sample	Country	Main Findings	Ref.
1	Beef patties	Facial expressions (FaceReader™ software- version 8, Noldus Information Technology, Wageningen, Netherlands).	N = 75. 22 to 76 yo. 48% female. From the University of Melbourne’s staff and students. Consumers.	Australia	Age-related differences were reported. Younger people exhibited higher intensity for happy/sad/scared and lower intensity for neutral/disgusted.	[80]
2	Grilled Pork Meat	Facial expressions (Internally developed model programmed in Python 3.13 using DeepFace 0.0.91, face recognition and facial attribute analysis framework retrieved from GitHub (https://github.com/)- software versions are not mentioned in the source).	N = 10. 60% females. Trained panellists. No additional information was provided.	Serbia	Increasing pungency intensity was associated with more non-neutral emotional facial expressions during consumption.	[79]
3	Smoked ham	Facial expressions (FaceReader 4 software).	N = 30. Mean age 23 yo. 70% female. Consumers.	Poland	Across all ham samples, emotions of neutral and negative valence dominated, with emotional variation driven more by individual consumers than by product differences.	[81]
4	Rabbit	Facial expressions (FaceReader 8 software).	N = 10. 32 to 42 yo. 80% female. Trained panellists.	Lithuania	Overall emotional responses were acceptable across treatments.	[82]

#### 5.2.2. Type of Product and Ingredients

Based on Bender (2014) [83], Scoville heat units (SHUs) are “the number of units of sugar water needed to be added to a part of ground chilli before the ‘burn’ is no longer detectable”. When the SHUs in a sauce applied to grilled pork were increased, non-neutral facial expressions also increased. The highest-rated emotion observed when the SHU in the sauce was raised was sad [79]. In the evaluation of smoked hams of different qualities, [84] found that high levels of happiness and low levels of negative facial expressions could differentiate a high-quality product. However, it is worth noting that these differences were relatively small and did not explain a significant portion of the overall variability [81].

In the case of rabbit meat, different muscles or meat from various feeding materials were able to lead to distinct facial expressions and, consequently, evoke different emotions in consumers. Alternative feeding systems resulted in up to seven times higher levels of happiness [82]. However, it should be highlighted that although facial movements have been widely associated with specific emotional states and interpreted as “facial expressions”, substantial evidence suggests that these movements are not universally shared across cultures, contexts, or individuals and may produce contradictory results [84].

### 5.3. Studies Measuring Emotional Responses to the Consumption of Plant-Based Alternatives or Combinations of Meat and Plant-Based Products

To the authors’ knowledge, only a few research papers have explored the emotions evoked during the consumption of PBMAs, highlighting a gap in the literature in this area. Some of these studies evaluated only PBMAs, while others examined them in combination with conventional meat products. A clear observation is the dominance of valence in emotional evaluations, regardless of whether PBMAs are presented alone or alongside conventional meat products. When both meat and plant-based cooked ham were evaluated together, most emotional differences were expressed through positive and negative valence; meat products were associated with emotions of positive valence, whereas PBMAs were linked to emotions of negative valence [85]. Interestingly, when PBMAs were evaluated alone (without comparisons to actual meat-based products), those similar to meat were associated with emotions of positive valence (e.g., satisfied, happy, hopeful, pleasant, and amazed), while those with low or no similarity to meat were associated with emotions of negative valence (e.g., dissatisfied, disappointed, uncertain, and unhappy) [9]. Similarly, another study reported that a PBMA with high similarity to conventional meat evoked more emotions related to surprise compared to the blended and pea-protein burgers [86].

**Table 3:** Studies on emotions elicited by consumption of plant-based meat alternatives and studies combining meat and plant-based products. Some studies may also include other products that fall outside the scope of this review and are not discussed here.

	Product	Technique/Outcome Measured	Sample	Country	Main Findings	Ref.
1	Plant-based and meat-based burgers	EmoSensory^®^ profile. 5-point rating [65]	N = 97. Mean age 27 yo. 36% females.	Belgium	Emotional responses were not affected by product information.	[66]
2	21 plant-based meat alternatives	They used an emotion lexicon developed and published previously to assess emotional response to plant-based burger patties [62]. CATA *.	N = 140. 25 to 45 yo. 74% females. Consumers.	New Zealand	Plant-based meat alternatives of high similarity to meat were associated with emotions of positive valence, while those with low similarity elicited emotions of negative valence.	[9]
3	Plant-based and meat-based burgers	A list of 11 statements associated with different emotions. CATA.	N = 177. 18 to over 65 yo. 67% females and 0.56% other. Consumers.	United States	Discrimination among products based on emotional perception increased when information was provided.	[86]
4	Meat and plant-based cooked ham	EsSense25. CATA.	N = 120. 18 to over 65 yo. 56% female. Consumers.	Ireland	Information provision increased emotions of positive valence and reduced emotions of negative valence for the nitrite-free ham but not the meatless product.	[85]

* CATA: Check-All-That-Apply.

When PBMAs were evaluated together with conventional products and meat-based healthier alternatives, several interesting observations emerged. For example, in a study including conventional, nitrite-free, and plant-based cooked ham, the conventional product, which consumers were familiar with, was associated with emotions of high valence but low arousal, such as calm, loving, and free. In contrast, the nitrite-free cooked ham was associated with emotions of both high valence and arousal, such as pleasant and joyful. The PBMA, on the other hand, was linked to emotions of high arousal and low valence, such as worried and disgusted, but also adventurous [85]. In another study, differences were observed depending on the product the PBMA was designed to imitate. Chicken-style PBMAs were associated with terms like hopeful, calm, neutral, and bored; beef-style products with emotions such as suspicious and disgusted; and chorizo-style sausages with emotions like energetic, curious, and adventurous [9].

### 5.4. The Effect of Context and Information

When individuals were asked to imagine themselves eating beef in a barbecue context, they tended to rate emotions of positive valence more highly. Emotions like joy and interest were significantly affected by this context [64]. The colour of food packaging could also be a significant factor affecting our emotions, impacting not only our food choices but also our emotional experiences during consumption. For example, red packaging, often used for hamburgers, was able to evoke emotions, during subsequent consumption, such as feeling wild, reassured, enthusiastic, and active. On the other hand, evaluation of products in white packaging tended to induce emotions of suspicion, while green packaging was able to evoke emotions such as calm, peacefulness, and disappointment. Interestingly, as consumption progressed, a balance was restored with similar emotions evoked by all products [72].

Moreover, when consumers were informed that the meat they are consuming is obtained from a well-known breed, it was observed that they tended to score it significantly higher for emotions such as enthusiasm, satisfaction, and pleasantness. Simultaneously, they scored significantly lower for emotions like worry, disgust, aggression, and boredom [63]. Providing information about the health benefits associated with a meat product was also able to influence the emotions it evokes. In such cases, emotions like active, interested, enthusiastic, and free tended to increase, while emotions of negative valence like worry tended to decrease [67]. Similarly, health-related claims regarding sodium reduction or elimination in roasted chicken increased scores for the emotion satisfied and lowered the scores for emotions of negative valence such as worried and unsafe [77].

When it comes to studies including PBMAs, a study on cooked ham reported only a few differences in the emotional perception of the products. That study included a conventional, a nitrite-free, and a plant-based sample (made with mycoprotein). After consumers were informed about the health risks associated with the conventional product and the health or environmental benefits of the nitrite-free and plant-based alternatives, only the nitrite-free product was associated with significantly higher happy and enthusiastic emotions, while consumers felt less disgusted [85]. Additionally, in another study where meat and plant-based burgers were presented either blindly or with information about their composition, consumers in the informed condition associated them with a greater range of emotions. However, no significant differences were observed within each burger type [86].

### 5.5. Emotions and Linking

Research on beef, ham, and chicken products showed that emotions of positive valence were associated with increased overall liking among consumers. In the case of beef, sensory attributes and hedonic scores were closely associated with a range of emotions of positive valence, including joyful, excited, pleasurable, glad, good, pleased, comfortable, happy, satisfied, pleasant, and positive feelings [64]. Several studies highlighted specific emotions of positive valence that were significantly related to increased liking. These emotions included feeling active, good, enthusiastic, happy, interested, pleasant, and satisfied. Conversely, emotions of negative valence like bored, guilty, disgusted, and worried have been linked to reduced liking [63]. In dry-cured hams, the liking was positively associated with emotions like intense, authentic, pleasant, and desirable, while negatively associated with the feeling of dissatisfaction [71]. Last, when it comes to products with novel formulations, liking was positively associated with emotions like satisfied, pleasant, good, and happy, and negatively with mild [78].

The link between emotions and liking was also influenced by the provision of information to consumers. In a study with meat- and plant-based burgers, under the blind condition, the main drivers of liking were similar across all burgers and were primarily satisfied, surprised, and gratified/rewarded. In the informed condition, however, additional emotions emerged as important drivers. For the meat-based burger, “happy memories of childhood” contributed to liking, while for the plant-based alternative that were similar to meat, “full of energy/reinvigorated” was among the top drivers. Conversely, decreases in liking were generally mainly associated with emotions of negative valence such as worried, disgusted, and disappointed [86].

## 6. General Considerations and Hypothesised Mechanism

Research on emotions in relation to meat consumption and reduction highlighted the role of meat in shaping shared identities, whether national, cultural, or related to constructions of masculinity, creating emotional bonds and affective economies. Within these groups, individuals emotionally respond to meat reduction strategies differently. Some perceive such strategies as a threat to their culture and sense of belonging, while others view them as moral considerations reflecting their values. Disgust is the most commonly studied emotion of negative valence in relation to meat consumption. The conflicting emotions associated with meat consumption can create cognitive dissonance, as consumers attempt to maintain internal consistency among their beliefs, attitudes, knowledge, and values. However, research shows that actual meat consumption triggers psychological processes that help regulate the emotion of negative valence associated with eating animals.

Emotional responses to meat consumption vary across individuals and are influenced by several factors. To date, there are several studies evaluating the emotional response to meat and only few to PBMAs. During meat consumption emotions of mainly positive valence are evoked, though different types, cuts, or formulations can elicit different responses in both valence and arousal. In contrast, PBMAs, when evaluated alongside conventional meat products, tend to evoke emotions of negative valence. Similarity to meat though is an important element as, interestingly, meat-like alternatives can elicit more emotional responses of positive valence. In both cases, meat and plant-based alternatives, emotional responses are also shaped by the consumption context and the information available to consumers. Emotions of positive valence are generally associated with increased liking for meat products, while emotions of negative valence are associate with decreased liking.

It should be noted, however, that the evidence base remains highly heterogeneous. The included studies differ in product types, evaluation context, participant characteristics, cultural setting, use of explicit versus implicit emotional measures, and whether PBMAs were assessed independently or in comparison with meat products. Moreover, although implicit methods may easier detect negative emotional responses, facial expression data should be interpreted cautiously because facial movements do not map reliably onto discrete emotional states across individuals, contexts, and cultures.

The differences observed in consumers’ emotional responses to meat products from different animal sources, formulations, or other attributes highlight the importance of the tasting experience in shaping emotional responses. These differences also indicate that the discourse on meat reduction, when discussed in general terms without specifying the type or characteristics of meat, overlooks important nuances in how consumers emotionally experience meat. It is evident that, although certain emotions are anticipated based on pre-existing beliefs, knowledge, and moral considerations, the actual consumption experience can strongly influence the emotions ultimately felt by consumers.

**Figure 1:**
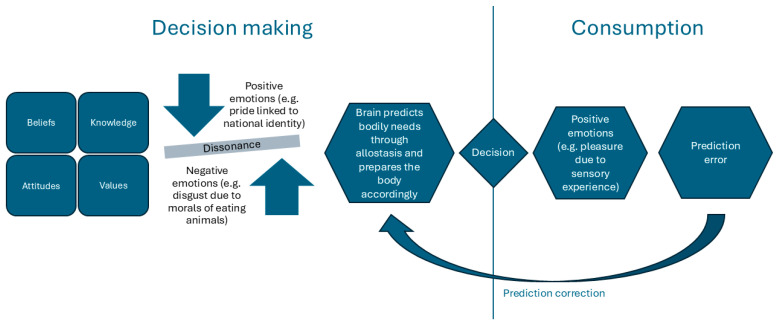
A conceptual framework (Consumption–Emotion–Update-CEU) of the role of emotions evoked during meat consumption in consumers’ decisions to consume or reduce meat.

Figure 1, based on the theory of constructed emotion, illustrates a conceptual framework on the role we hypothesise that emotion evoked during actual meat consumption plays in consumers’ decisions to consume or reduce meat. This is not presented as a universal model of decision-making, but given that much of the literature on emotions and meat reduction overlooks the actual consumption experience, Figure 1 aims to illustrate how emotions evoked during meat consumption can influence subsequent decisions. During decision-making, individuals may experience conflicting emotions shaped by their beliefs, attitudes, knowledge, and values. For example, they may feel pride linked to national identity but also disgust due to moral concerns about eating animals or worry about health impacts from modern production systems. Such emotional conflict can generate cognitive dissonance, creating psychological discomfort that individuals attempt to reduce. Part of this dissonance may be alleviated during consumption. Empirical studies, as seen in Section 4, report that actual meat consumption elicits mostly emotions of positive valence, while the consumption of PBMAs elicits emotions of negative valence.

Therefore, from the perspective of the theory of constructed emotion, we hypothesise that during the decision-making stage, before consumption, the brain predicts bodily needs through allostasis and prepares the body accordingly. When the actual consumption experience does not match the positive or negative valence predicted on the basis of moral values or previous experiences, this mismatch may generate a prediction error, prompting the brain to update its model. We conceptualise this mechanism as the Consumption–Emotion–Update (CEU) framework. When it comes to meat consumptions, this updating may prevent negative beliefs about meat consumption from dominating. Conversely, with plant-based alternatives, which often do not possess the expected profile, during consumption, the opposite pattern may occur, reinforcing negative expectations.

It should be highlighted that the proposed mechanism describing how consumption-evoked emotions may contribute to future choice behaviour is a hypothesised mechanism, and direct longitudinal or behavioural evidence remains limited.

## 7. Conclusions

This review provides insights into the complex relationships between emotions, meat consumption and reduction, and the consumption of PBMAs. Drawing on practice theory and the theory of constructed emotion, it proposes a conceptual mechanism on how emotions elicited during the actual consumption of meat and PBMAs may influence subsequent intentions and decisions related to meat consumption or reduction.

The evidence reviewed suggests that emotions associated with meat and its alternatives are shaped by prior experiences, social practices, cultural meanings, and the immediate consumption context. While public discourse surrounding meat often evokes both positive and negative emotions, actual meat consumption is more commonly associated with positively valanced emotional experiences. In contrast, PBMAs tend to elicit emotions of positive valence to a lesser extent, particularly when their sensory characteristics differ substantially from those of meat. These findings highlight the importance of examining emotions during consumption, rather than relying solely on attitudes or intentions, to better understand food choice behaviour.

Although the Consumption-Emotion-Update (CEU) framework requires empirical validation, it offers a tool for future research on the role of emotions in dietary change. A deeper understanding of how emotions are elicited during food consumption may help explain why intentions to reduce meat consumption do not always translate into behavioural change.

From a practical perspective, the findings suggest that interventions promoting meat reduction and the adoption of PBMAs should pay greater attention to the consumption experience itself and the emotions related to it. Ultimately, understanding how consumption-evoked emotions shape food choices can contribute to the development of more effective products, interventions, and policies aimed at supporting healthier and more sustainable dietary patterns.

## Figures and Tables

**Table 1:** Studies on emotions elicited by consumption of meat, measured explicitly.

	Product	Technique/Outcome Measured	Sample	Country	Main Findings	Ref.
1	Beef	Italian version of EsSense25 (intensity from 1 to 5) (good-natured and joyful excluded). CATA *.	N = 93. 18 to 65 yo. 51% females.	Italy	The “Only from the Italian Simmental” label elicited more emotions of positive valence and less of negative valence than the conventional label.	[63]
2	Beef	In-study development of emotions vocabulary in Portuguese (developed with the help of EsSense). RATA ** (1 slightly applicable to 5 very applicable).	N = 77. 18 to 65 yo. 61% females.	Brazil	Rump cap was associated with more emotions of positive valence, while outside flat elicited more emotions of negative valence.	[64]
3	Meat burgers	EmoSensory^®^ profile. 5-point rating [65]	N = 97. Mean age 27 yo. 36% females.	Belgium	Emotional responses were not affected by product information.	[66]
4	Beef Rib-eye steaks	EsSense profile. RATA.	N = 150. 18 to 60 yo. No additional information was provided.	United States	Health benefit information increased emotions of positive valence, particularly “interested” and improved emotional profiles across all beef steak types.	[67]
5	Beef (Angus beef, lean in fat) meatballs	‘Emotions in food experience’ [68]: a shortened version (18 of the 22 terms—shame, sadness, anger, jealousy were excluded). RATA (1: not at all, to 5: strongly).	N = 101. 25 to 65 yo. No data on gender.	Australia	Consumers generally showed positive emotional and affective responses toward meat products, produced with recycled water.	[69]
6	Chicken (with two traditional spices)	15 emotional terms. CATA.	N = 218. 133 Chinese and 85% Pakistani. 20 to 30 yo. 63% females (Chinese) and 26% females Pakistanis)	China	Chicken with spice blends elicited emotions of positive valence, while plain chicken was associated with emotions of negative valence	[70]
7	Dry-cured hams	In-study development of emotional lexicon (they propose a method). TDE ***.	N = 15. 20 to 50 yo. 73 females. Staff of the University of Extremadura.	Spain	Iberian ham evoked more emotions of positive valence, while Serrano and Curado ham elicited more emotions of negative or neutral valence.	[71]
8	Hamburgers	12 emotions in Brazilian Portuguese generated with focus group with the help of EsSense. TDE.	N = 92. Mean age 22 yo. 58% females.	Brazil	Packaging colour influenced the emotional responses during the first stages of consumption.	[72]
9	Chinese blanched chicken	10 emotion terms selected from the literature [73]. CATA.	N = 61. 18 to 40 yo. 56% females.	China	Different chicken parts evoked distinct emotional responses, with breast meat associated with more favourable emotional perceptions.	[74]
10	Pork	Consumers were asked to evaluate how well they found that the samples fitted to some attributes (i.e., harmonic, complex, well-known, delicious, boring, traditional, summer-like, Nordic and fullness) on a 15 cm (“not at all” to “very much”).	N = 44. 26 to 69 yo. 64% female.	Denmark	Consumers showed more positive emotional associations with the alternative than with the traditional crossbreed.	[75]
11	Frankfurter sausages	EsSense25. CATA.	N = 120. 18 to 55 yo. 63% females.	Brazil	Significant differences were found in terms of emotional perception, among different sausages types.	[76]
12	Roasted chicken	A selection of emotions (i.e., good, happy, interested, pleased, satisfied, unsafe, and worried) were chosen based on the online survey. Rated on a 5-point intensity scale.	N = 250. No additional information was provided.	Honduras	Higher potassium chloride substitution reduced emotions of negative valence, while positive emotions were key predictors of purchase intent.	[77]

* CATA: Check-All-That-Apply, ** RATA: Rate-All-That-Apply, and *** TDE: Temporal Dominance of Emotions.

## Data Availability

No new data were created or analyzed in this study.
